# Radiopharmaceuticals for Breast Cancer and Neuroendocrine Tumors: Two Examples of How Tissue Characterization May Influence the Choice of Therapy

**DOI:** 10.3390/cancers12040781

**Published:** 2020-03-25

**Authors:** Alberto Signore, Chiara Lauri, Sveva Auletta, Michela Varani, Livia Onofrio, Andor W. J. M. Glaudemans, Francesco Panzuto, Paolo Marchetti

**Affiliations:** 1 Nuclear Medicine Unit, Department of Medical-Surgical Sciences and of Translational Medicine, “Sapienza” University of Rome, 00189 Rome, Italy; chialau84@hotmail.it (C.L.); sveva.auletta@hotmail.it (S.A.); varanimichela@gmail.com (M.V.); livia.roma@live.it (L.O.); 2 Department of Nuclear Medicine and Molecular Imaging, University of Groningen, University Medical Center Groningen, 9700 Groningen, The Netherlands; a.w.j.m.glaudemans@umcg.nl; 3 Digestive Disease Unit, AOU Sant’Andrea and ENETS Center of Excellence, 00189 Rome, Italy; francesco.panzuto@uniroma1.it; 4 Oncology Unit, Department of Clinical and Molecular Medicine, “Sapienza” University of Rome, and IDI-IRCCS, 00189 Rome, Italy; paolo.marchetti@uniroma1.it

**Keywords:** nuclear medicine, PET/CT, radiopharmaceuticals, therapy decision making, treatment response, personalized medicine, breast cancer, neuroendocrine tumors

## Abstract

Molecular medicine has gained clinical relevance for the detection and staging of oncological diseases, to guide therapy decision making and for therapy follow-up due to the availability of new highly sensitive hybrid imaging camera systems and the development of new tailored radiopharmaceuticals that target specific molecules. The knowledge of the expression of different receptors on the primary tumor and on metastases is important for both therapeutic and prognostic purposes and several approaches are available aiming to achieve personalized medicine in different oncological diseases. In this review, we describe the use of specific radiopharmaceuticals to image and predict therapy response in breast cancer and neuroendocrine tumors since they represent a paradigmatic example of the importance of tumoral characterization of hormonal receptors in order to plan a tailored treatment. The most attractive radiopharmaceuticals for breast cancer are 16α-[^18^F]-fluoro-17β-estradiol for PET assessment of the estrogen expression, radiolabeled monoclonal antibody trastuzumab to image the human epidermal growth factor receptor 2, but also the imaging of androgen receptors with [^18^F]-fluorodihydrotestosterone.

## 1. Introduction

In the last decades, huge progress has been made in the field of tumor microenvironments and, in particular, in the matter of tumor immunology; therefore, many efforts are constantly being directed towards molecular imaging in several diseases. This new approach is gradually revolutionizing the diagnostic and therapeutic strategies in oncology being able to provide an in vivo “histological characterization” of different cell types, subtypes and molecules involved in pathological processes, thereby aiming to plan a personalized therapy and to follow-up its efficacy. A correct and prompt diagnosis is crucial, indeed, for both patient and referring clinician, since the more promptly the diagnosis is settled, the earlier and more appropriate therapy can be started, leading to a better response and less morbidity and mortality. Traditional radiological imaging techniques, focus on macroscopic anatomic changes that usually occur at advanced stages of the disease, and usually show a moderate specificity in demonstrating inflammatory phenomena.

On the other hand, nuclear medicine (NM) techniques offer the possibility to identify functional changes even before the clinical onset of the disease, thereby changing the diagnostic approach and therapy of many pathologies [1]. According to the complexity of mechanisms involved in disease processes, different radiopharmaceuticals, exploring different aspects of the disease, may be used. The availability of new highly sensitive hybrid imaging camera systems, such as single-photon emission computed tomography/computed tomography (SPECT/CT), positron emission tomography/computed tomography (PET/CT) and positron emission tomography/magnetic resonance imaging (PET/MRI), that combine functional information, provided by the radiopharmaceutical, with the anatomical data provided by CT or MRI, together with the development of new tailored radiopharmaceuticals targeting specific molecules, has become clinically relevant for therapy decision making and follow-up [2,3]. Many drugs and radiopharmaceuticals have been developed and tested in several clinical and preclinical trials for imaging tumor microenvironment and they may also be used, in genetically susceptible subjects, for early diagnosis in the pre-clinical phase of diseases, for staging, for detection of disease relapse, and for radio-guided surgery. 

The majority of target molecules identified so far are functional receptors, cytokines or cytokine receptors. To specifically image these molecules, we can use two different approaches: to radiolabel the artificial ligand of the receptor molecule or to use a monoclonal antibody (MoAb) directed against the target molecule. Radiolabeled receptor ligands (such as cytokines) can provide an excellent solution for molecular imaging due to their short plasma half-life, low background uptake, low accumulation in excretory organs and fast blood kinetics; however, they may have side effects due to their biological action. On the other hand, radiolabeled MoAbs are well known for their high specificity against target molecules and offer exciting possibilities for the selection of candidate patients for the therapy. Many MoAbs are now commercially available, in some cases also as a kit for easy radiolabeling. For example, it is possible to study angiogenesis in the tumor environment with the Bevacizumab, a MoAb directed against the vascular endothelial growth factor (VEGF), that blocks the creation of new vessels thus reducing blood supply to the tumor. This drug can be radiolabeled with 99m-technetium (^99m^Tc) or 111-indium (^111^In) or other isotopes for SPECT studies [3,4,5]; however, it can also be used for PET imaging, coupled to the radionuclide 89-zirconium (^89^Zr). This radiopharmaceutical could be used to both visualize angiogenesis and to monitor the effect of anti-angiogenetic treatments in different neoplasms [6,7,8,9,10].

In this review article, we highlight the capability of functional imaging to show receptor expression on cancer cells, with different radiopharmaceuticals, that can be possible therapeutic targets. Several examples could be mentioned in this regard, as shown in Table 1, but we concentrated on two examples: breast cancer and neuroendocrine tumors.

In breast cancer, hormonal receptors for estrogen (ER), progesterone (PR) and androgens (AR) can be used as a target for non-invasive whole-body evaluation of the hormonal status and to predict the response of cancer to endocrine treatment. At this moment, the most attractive radiopharmaceuticals are 16α-[^18^F]-fluoro-17β-estradiol (FES) for PET imaging of the ER expression [11] and the radiolabeled (both for SPECT and PET) MoAb trastuzumab to image the human epidermal growth factor receptor 2 (HER2) [11,12,13,14].

Neuroendocrine tumors (NETs) represent another situation in which NM may play a role in tumoral characterization by imaging somatostatin receptor (SSR) expression, with both SPECT and PET radiopharmaceuticals [15]. OctreoScan™ ([^111^In]-DTPA-penteoctreotide) was one of the first radiopharmaceuticals for imaging well-differentiated NETs that express SSR, particularly subtypes two and five, and this imaging modality is still widely used when PET is not available [16,17]. Due to its poor spatial resolution, long time acquisition and lower sensibility and specificity, imaging of SSR with 1,4,7,10-Tetraazacyclododecane-1,4,7,10-tetraacetic acid (DOTA)-somatostatin analogs radiolabeled with 68-gallium (^68^Ga), may be attempted. They are able to detect and achieve an accurate diagnosis and to guide the best therapy choice such as surgery, chemotherapy or peptide receptor radionuclide therapy (PRRT) [18,19].

The aim of this review is summarize the state-of-the-art molecular imaging for oncological diseases, particularly breast cancer and NETs, describing the currently existing NM imaging techniques and radiopharmaceuticals able to help in therapy decision making and to evaluate therapy efficacy (Table 1).

## 2. Breast Cancer: from Conventional Approaches to Molecular Imaging with Human Epidermal Growth Factor Receptor 2, Estrogen and Androgen Receptors

Breast cancer is still one of the most common cancers in the Western world. The primary goal for the successful treatment of breast cancer is the precise characterization and definition of the extent of disease. Therefore, staging is a crucial moment for the management of these patients. The oncologist is constantly asked to choose the best diagnostic approaches able to provide the most comprehensive overview of a patient’s status, sparing time and resources and aiming to promptly start the most appropriate treatment. Since a single imaging modality able to do this still does not exist, the best approach derives from the combination of different techniques that provide complementary information about the same problem. In clinical practice, conventional imaging with mammography, ultrasound (US) and breast MRI are commonly used for staging local disease extent, whereas whole-body CT, PET/CT with 2-deoxy-2-[18F] fluoro-D-glucose ([^18^F]-FDG PET/CT) and bone scans are useful in systemic staging. 

In particular, from an NM point of view, the role of [^18^F]-FDG PET/CT in staging, follow-up and therapy response assessment in breast cancer is well known and widely adopted. Indeed, this modality shows an added value over conventional radiology for the evaluation of extra-axillary lymph nodes (internal mammary or supraclavicular) and for distant metastasis, thus providing important information for planning surgical strategy and for correct design of the radiation therapy fields. However [^18^F]-FDG PET/CT is not indicated in patients with clinical stage I breast cancer, since distant metastases are rarely observed in this early-stage disease and cannot replace conventional diagnostic approaches in the identification of primary tumors because of the high number of false positives (fibroadenomas, flogistic processes) and false negative results, especially in the presence of small lesions [20]. In breast cancer restaging and therapy follow-up [^18^F]-FDG PET/CT is also widely used in combination/alternative to conventional radiology showing very high accuracy in both loco-regional recurrence and distant metastasis detection [21]. The low specificity of [^18^F]-FDG represents a limitation of its use immediately after surgery and/or radiotherapy, since the high uptake observed in flogistic processes cannot allow excluding the concomitant presence of active disease. Another possible limitation of this imaging modality resides in the evaluation of osteoblastic bone metastases that usually show lower avidity for [^18^F]-FDG compared to lytic metastasis, and therefore, bone scintigraphy should be added.

Currently, the most commonly used criteria for defining response on treatment are the RECIST (Response Evaluation Criteria for Solid Tumors) v. 1.1 [22,23]. These criteria are based on anatomic measurements: tumor response if the diameter of the tumor has decreased ≥ 30%, and tumor progression if there is growth ≥ 20% or new lesions. However, these criteria can only be applied in measurable lesions, which is often not the case in breast cancer patients, since bone metastases are the most common site of distant metastases [24]. So, for therapy evaluation, it would be valuable to have a tool that allows (early) response evaluation in patients with bone metastases [25]. PET response criteria in solid tumors (PERCIST) have been proposed to assess the metabolic response to therapy by [^18^F]-FDG-PET but some aspects still remain unresolved [26]. Therefore, alternative and more tailored approaches for diagnosis and therapy monitoring are needed.

For metastatic breast cancer, endocrine agents, monoclonal antibodies, and chemotherapy given sequentially as mono or combination therapy, are the mainstay of therapy. In the past, a one-size-fits-all approach (a standard approach, not tailored to the individual characteristics of the tumor and metastases) was used for these patients, leading to inefficient therapeutic strategies with off-target side effects. In the last years, a shift is occurring in treatment towards precision medicine, which aims to treat an individual patient with tailored drugs based on characteristics of the tumor and its metastases. Molecular imaging can help in this tailored individual treatment strategy.

In precision medicine, for staging, therapy prediction and therapy response measurements, knowledge of the level of expression of different receptors on the primary tumor but also on metastases throughout the body are important for both therapeutic and prognostic purposes. It is also well known that the expression levels may switch in time, e.g., a metastasis without any receptor overexpression in the beginning may become positive within the next months or years and vice versa. Most important receptors, for which therapeutic drugs and molecular imaging techniques are available, are the ERs, the HER2 and AR. We will now describe which molecular imaging tracers are available to visualize the expression of these receptors and the current standing of these tracers in clinical practice.

### 2.1. Imaging of Estrogen Receptor (ER) Status

ER is overexpressed in approximately 70% of breast cancer patients, making targeted endocrine therapy an attractive treatment option, especially in the metastatic setting. Three classes of endocrine treatment are available: selective modulators that competitively bind to the ER (tamoxifen), aromatase inhibitors that have an indirect effect on the ER by inhibiting the conversion of androgens into estrogens and selective ER down-regulators such as fulvestrant, that degrade the ER [27].

Currently, ER status is determined by immunohistochemistry on the primary breast tumor, and, if possible, mostly on one metastasis. However, more knowledge has become available about heterogeneity between metastases, the discordant expression between primary and metastases and changes in expression over time that occurs in a large group of patients (up to 40%) [28] Furthermore, a biopsy of metastases is not always feasible due to location, may cause complications and can lead to sample errors, especially in metastases with intra-humoral heterogeneity of the ER. Up-to-date knowledge of the ER status, not only of the primary tumor but also of all metastases, is important for prognosis, prediction of therapy and for treatment decision making.

Molecular imaging by [^18^F]-FES-PET provides non-invasive information of the ER status of all metastases throughout the body of the individual patient. Furthermore, this imaging technique can also give insights about the ER binding of endocrine ER modulators or down-regulators by analyzing the differences in ER status before and during therapy. Earlier preclinical studies showed that there is a good correlation between [^18^F]-FES uptake and ER expression by immunohistochemistry [29,30], and that the level of [^18^F]-FES uptake predicts the response to hormonal therapy [11]. Recently, recommendations for patient preparation, scan acquisition, analysis and interpretation were published [31].

Multiple studies with [^18^F]-FES-PET in patients with breast cancer have been performed, with an overall sensitivity of 84% and an overall specificity of 98%^24^ in those studies that correlated with [^18^F]-FES-PET results directly with immunohistochemistry. For therapy prediction, a cut-off Standard Uptake Value_max_ (SUV_max_) of 1.5 can be taken to select patients. In general, a SUV_max_ lower than 1.5 predicts failure to respond to endocrine therapy, whereas a SUV_max_ higher than 1.5 predicts response to therapy. This threshold is based on several published studies [11,28,32,33], however, as it also stands for [^18^F]-FDG, the role of semiquantitative analysis of the uptake by using SUV is not universally recognized because it depends on several factors. In particular, time from radiopharmaceutical injection and scan, patient size, fasting conditions and other technical issues are the main sources of variability and, therefore, they should always be considered in the evaluation of SUV especially in the follow-up studies or when comparing different scans acquired in different centers [34].

**Figure 1:**
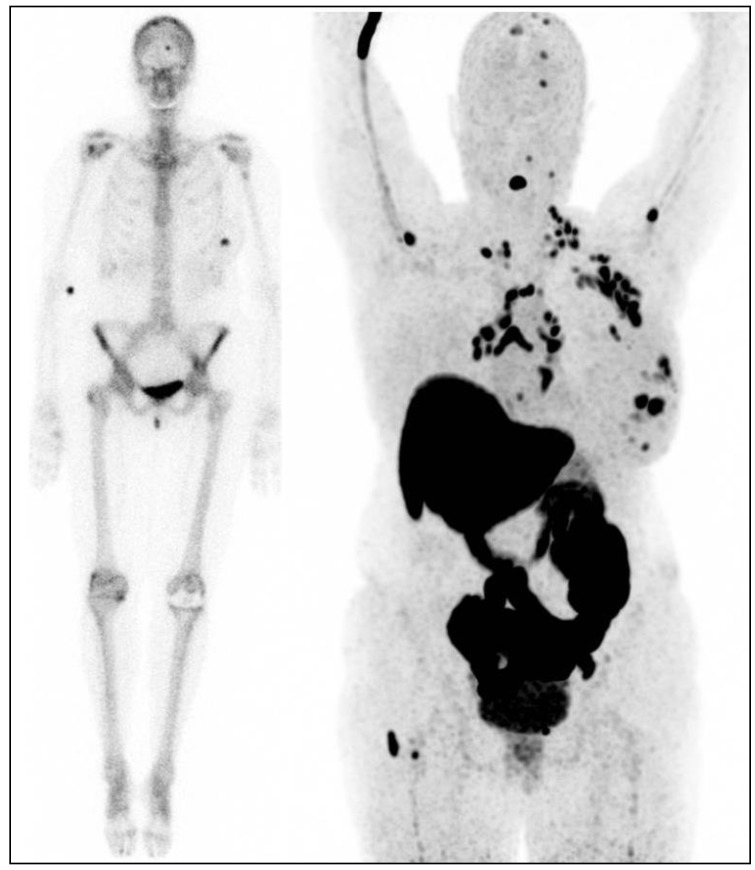
Female patient, known with the estrogen receptor (ER) positive breast cancer in her left breast. Left image: bone scan for dissemination: uptake in the primary tumor in the left breast, and uptake in the skull (metastasis or bone island?). Right image: [^18^F]-FES-PET with multiple lesions in the left breast, multiple ER positive lymph node metastases (axillae, clavicular regions, neck, mediastinum and hili) and multiple ER positive bone metastases (skull, spine, left humerus, right femur).

**Figure 2:**
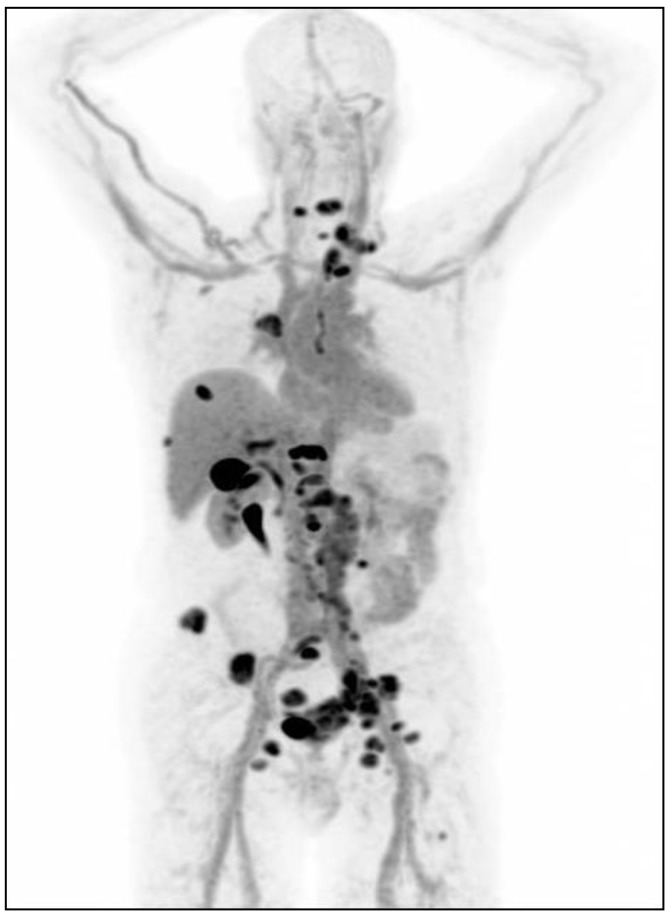
Example of [^18^F]-FDHT-PET in a patient with metastasized prostate cancer: lymph node metastases in the neck, right hilus, retroperitoneal, para-iliacal and inguinal, bone metastases in the spine, ribs, pelvis and left femur.

[^18^F]-FES-PET can also be used as a diagnostic tool in patients that present with a clinical dilemma, that cannot be solved by conventional imaging and/or when a biopsy is not feasible. For example, patients with known ER positive breast cancer in the past who now present (1) with lesions on CT, MRI, [^18^F]-FDG-PET, bone scintigraphy, etc., in which still a differential diagnosis exists (Figure 1), (2) with a new different primary tumor and lesions that could be metastases of the earlier breast cancer or of the new tumor, (3) with a solitary lesion of which biopsy is not feasible due to location and knowledge of the ER status is important for therapy decision making, etc.

A study in 33 patients that underwent [^18^F]-FES-PET imaging for clinical dilemmas showed that [^18^F]-FES-PET supported therapy decisions by improving diagnostic understanding of the referring clinician in 88% of the cases, and a change in therapeutic strategy was reported in 48% of the patients [35]. Other studies showed the added value of [^18^F]-FES-PET in staging and therapy decision making in patients with disseminated lobular breast cancer, a type of breast cancer that is often difficult to detect with conventional imaging because they tend to grow less cohesively than other breast cancer types [36].

In progressive patients, despite several lines of anti-hormonal therapy, [^18^F]-FES-PET can also be used to provide information if metastases still express the ER, thereby providing a rationale for another line of anti-hormonal therapy (if the [^18^F]-FES-PET is positive) or for a switch to another treatment option, in case of a negative [^18^F]-FES-PET [31].

The next step is the use of ^18^F-FES-PET in therapy response and decision making by measuring the response to starting endocrine therapy. For example, serial [^18^F]-FES-PET imaging was performed to evaluate the effect of 500 mg fulvestrant on FES uptake in patients with metastatic breast cancer. [^18^F]-FES-PET was used to study the ability of fulvestrant to reduce ER availability in the individual patient and to correlate this with treatment response. A reduction of [^18^F]-FES uptake, between baseline and the four weeks’ scan, larger than 75% was significantly associated with clinical benefit. However, fulvestrant did not block the ER completely in all patients and some patients may benefit from higher doses of fulvestrant [37].

Normally, therapy evaluation is performed by repeated CT scans which may take several months before therapy effects can be reliably measured. [^18^F]-FES-PET can provide this prediction of treatment response much earlier, and may even play a role in the decision making to adjust the dose of the intended treatment or to switch to another treatment option in the individual treatment. This strategy was confirmed by Yang and colleagues. They showed the value of [^18^F]-FES imaging to predict response to neoadjuvant chemotherapy, especially when correlated with [^18^F]-FDG uptake [38]. For the evaluation of new ER targeting drugs, [^18^F]-FES-PET can be used to determine changes in ER status and tumor responses during treatment and with different dosage schedules of the new drug [39].

[^18^F]-FES-PET mainly visualizes the ERα status of the tumor and metastases. Despite the fact that gynecological tumors such as ovarian cancer and endometrial sarcoma mostly express ERβ, [^18^F]-FES-PET was also able to reliably assess the ER status in these tumors [40]. Nevertheless, attempts are on-going to produce specific ERβ tracers [41].

### 2.2. Imaging of the Human Epidermal Growth Factor Receptor 2 (HER2)

HER2 is overexpressed in the minority of breast cancer patients, approximately 20–25% [42]. It is, however, a critical biomarker since HER2 overexpression is associated with aggressive growth and poor prognosis [43]. HER2 positive breast cancer patients have a worse prognosis compared to ER positive breast cancer patients. The anti-HER2 monoclonal antibody trastuzumab is part of treatment in both the adjuvant as the metastatic setting of HER2 positive breast cancer [44].

Trastuzumab therapy is expensive and may lead to severe side effects, and therefore, adequate patient selection for this treatment is of invaluable importance. As for ER status, HER2 expression may change between the primary breast tumor and its metastases and also heterogeneity within and across lesions in the individual patients is well known [45,46]. It was also suggested that 10–15% of patients with HER2 negative primary breast cancer may still benefit from trastuzumab treatment due to the conversion of the receptor in time [47]. As for ER, up-to-date knowledge of the HER2 status of the primary tumor and all metastases is necessary to identify which patients may benefit from HER2-targeted treatment.

To provide non-invasive information of the HER2 status of all lesions within the body of an individual patient, first, the SPECT tracer [^111^In]-trastuzumab was developed, and at a later stage, was followed by the PET tracer [^89^Zr]-trastuzumab. ^89^Zr was chosen as radionuclide because of its half-life of 78.4 h, compatible with the biological half-life of trastuzumab. In a first proof-of-concept study in patients, the optimal scan day (four days after injection) and amounts of the cold and radiolabeled antibody were determined. Moreover, most metastatic lesions showed a good tracer uptake and, in two patients, unknown brain metastases were detected [14]. In another proof-of-concept study, [^89^Zr]-trastuzumab PET detected unsuspected HER2 positive metastases in patients with HER2 negative primary breast cancer [48], showing the heterogeneity between primary tumor and metastases.

The use of [^89^Zr]-trastuzumab PET is not as far advanced as the [^18^F]-FES-PET imaging technique. Work to define thresholds for declaring a lesion positive is right now on-going but still difficult because of the high uptake in liver and blood vessels. As a consequence, uptake of [^89^Zr]-trastuzumab in the metastases is also partly due to high perfusion and angiogenesis. At this moment, [^89^Zr]-trastuzumab PET is mainly used in research trials. Trastuzumab was recently also labeled as 64-copper (^64^Cu) which has a half-life of 12.7 h [49,50]. Comparative studies between ^64^Cu- and [^89^Zr]-trastuzumab are not available yet.

The added value for both [^18^F]-FES-PET and [^89^Zr]-trastuzumab PET in therapy prediction and selection in patients with metastasized breast cancer is right now evaluated in a multicenter study in the Netherlands, the “Imaging Patients for Cancer drug selecTion in metastatic breast cancer” (ClinicalTrials.gov Identifier: NCT01957332). By using these relevant PET scans, it is possible to visualize a tumor-specific profile for all metastatic lesions and demonstrate heterogeneity within the individual patient. The aim of this study is to optimize patient selection using molecular imaging and therapy improving therapy selection. Two hundred patients will be included in this study.

### 2.3. Imaging of the Androgen Receptor (AR)

Routinely, in breast cancer at immunohistochemistry, the ER, HER2 and PR statuses are determined, but not the AR. Relatively scarce information is available about the role of androgens and the AR in breast cancer. Several studies found, however, expression of the AR in 60–85% of breast cancers, and in some patients even at higher levels than the ER [51,52]. High circulating androgen levels in postmenopausal women were also found to be associated with an increased risk of developing breast cancer [53,54]. A possible anti-proliferative effect of AR stimulation and pathway activation in breast cancer is suggested [55].

AR therapy in patients with metastatic breast cancer was already applied a long time ago, but severe side effects resulted in the end of this therapy line. Recently, since several less toxic AR-targeted drugs became available and were approved by the Food and Drug Administration (FDA) for patients with prostate cancer, interest in evaluating the AR expression in breast cancer patients has been growing, especially in the triple-negative (ER, PR and HER2 negative) patients and in those patients that develop resistance to current breast cancer treatment possibilities. As is the case for ER and HER2 targeted therapy, the challenge is to administer AR-targeted drugs to the right patient, to increase the rate of successful therapy and to decrease the level of side effects.

**Figure 3:**
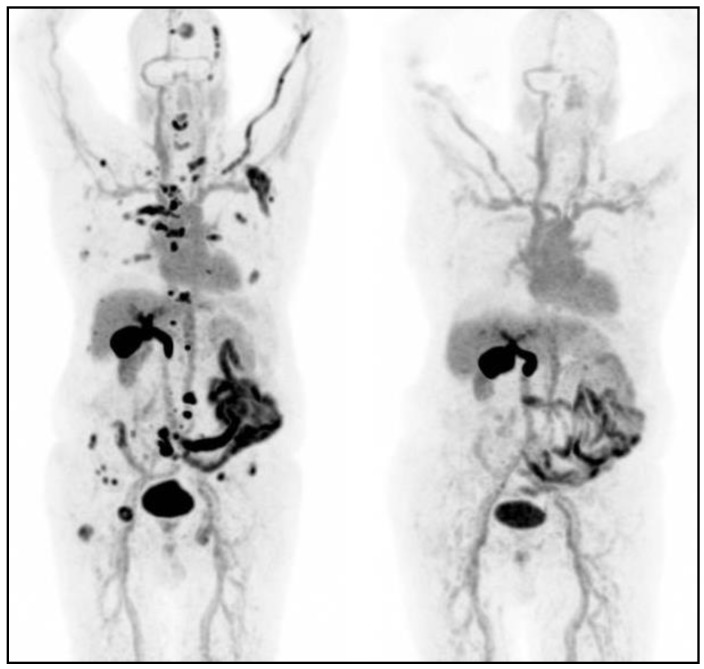
Example of [^18^F]-FDHT-PET in a patient with metastasized prostate cancer: lymph nodes and bone metastases before (left) and during treatment with Enzalutamide.

For the non-invasive visualization of the AR expression of tumors and metastases throughout the body, the PET tracer [^18^F]-fluorodihydrotestosterone (FDHT) is available (Figure 2 and Figure 3). This tracer is already studied in metastatic prostate cancer patients, where [^18^F]-FDHT uptake was found in the majority of metastases [56]. Furthermore, this uptake was found to be specific, since the uptake can be blocked by AR antagonists (flutamide and MDV3100) [57,58]. Several studies with [^18^F]-FDHT-PET for response prediction and therapy evaluation in patients with prostate cancer are on-going.

In breast cancer imaging, studies to visualize the AR expression are limited. Recently, one feasibility study was performed in 13 evaluable breast cancer patients. [^18^F]-FDHT uptake correlated well with AR expression levels in representative biopsies with a SUV_max_ cut-off of 1.94 leading to a sensitivity of 91% and a specificity of 100%. These results show the potential use of whole-body [^18^F]-FDHT-PET imaging for AR status [59]. Larger studies, however, are necessary to optimize the cut-off value for positivity. A study assessing the AR occupancy by the AR targeting drug bicalutamide by measuring the treatment-inducing change in AR availability with [^18^F]-FDHT-PET is on-going (ClinicalTrials.gov Identifier: NCT02697032). In the forthcoming years, [^18^F]-FDHT-PET has to be evaluated for its added value as a diagnostic tool (similar to [^18^F]-FES), and for therapy prediction and therapy response monitoring in both prostate and breast cancer.

### 2.4. Imaging Treatment Response

As reported above, delineating the tumor receptor profile has become extremely important for the staging and the restaging of cancers, as well as guiding the clinician in choosing the best therapeutic option. For breast cancer, several radiopharmaceuticals are available to study which receptors are mainly expressed and in which lesions’ sites, making molecular imaging an effective diagnostic tool, together with immunochemical analysis. The [^18^F]-FES-PET is a clear example of a non-invasive imaging modality that provides the ER status of the primary tumor and all metastases and, secondly, allows the evaluation of treatment response, monitoring the therapeutic efficacy (e.g., fulvestrant therapy in breast cancer patients) and therapy decision making in relation to the [^18^F]-FES-PET scan outcome. Therefore, having a clear clinical picture of tumor characterization of each patient would lead to a tailored therapy, both intra-patient and intra-lesion, combining different therapies.

## 3. Somatostatin Receptors as Targets for Imaging NETs

NETs are defined as malignancies, usually slow-growing, deriving from diffuse neuroendocrine cells that frequently involve lungs, gastrointestinal tract, pancreas and, less often, the autonomous nervous system. The European Neuroendocrine Tumor Society (ENETS) published several guidelines for the classification, diagnosis, prognosis and therapy of NETs [16,18,19,60,61,62].

According to the recent World Health Organization (WHO) classification and the ENETS guidelines, neuroendocrine neoplasia (NEN) may be divided into five categories: benign tumors, resectable tumors of probably benign behavior, resectable malignant tumors with/without regional nodal involvement, non-resectable tumors with/without nodal involvement and/or with/without liver and other metastases and familial tumors (Multiple Endocrine Neoplasia (MEN-1) Syndrome and von Hippel Lindau Disease) [61,63]. Furthermore, NEN classification follows a precise grading (G1, G2 and G3) that, together with the identification of metastases, constitutes the most important prognostic factor and allows distinguishing between well-differentiated NETs and poorly-differentiated neuroendocrine carcinomas (NEC). The grading of well-differentiated NETs and poorly-differentiated NEC is highly correlated to the histological evaluation of proliferation index, particularly the Ki-67 staining and, less often, the mitotic count [60,64]. In addition to histopathological analysis, imaging procedures, including radiological and NM methodologies, are of large utility for the diagnosis, staging (characterization of primitive and distant lymph node metastatic lesions), the unknown primary tumor detection, the therapeutic planning for patients with NETs expressing SSR and eligible for PRRT, therapy monitoring and the metabolic evaluation for patients with high-grade NETs.

Initially, OctreoScan™ was recommended as a first-line radiopharmaceutical for staging, diagnosis, follow-up of NETs and for the selection of patients with metastatic tumors for PRRT [19], but it is slowly being replaced by DOTA-peptides radiolabeled with ^68^Ga for PET imaging. 

Several studies have explored the role of [^68^Ga]-DOTA-peptides in the diagnosis and treatment management of NETs. Among them, DOTA-NOC has more favorable dosimetry and affinity, not only for SST2 and SST5 like DOTA-TOC and DOTA-TATE, but also for SST3. Some authors reported that [^68^Ga]-DOTA-NOC PET/CT influences the therapy decision in more than 50% of enrolled patients, particularly when PET and CT are discordant with higher accuracy compared to conventional imaging (83.4% vs. 74.3% in patients with primary NETs, 98.2% vs. 87.2% in patients with metastatic NETs, respectively, for [^68^Ga]-DOTA-NOC and CT) [65,66]. Furthermore, [^68^Ga]-DOTA-NOC PET/CT revealed to be better than [^111^In]-DTPA-octreotide scintigraphy because of clearer image resolution, lower background noise, higher specificity by detection of a higher number of lesions and better toleration by patients [67].

[^68^Ga]-DOTA-TATE has been investigated by many authors in comparison to conventional imaging (CT/MRI) or [^111^In]-pentetreotide/octreotide, reporting a change in patient management in the range of 32.8–70.6% [68,69,70,71,72,73,74], being more sensitive and accurate than [^111^In]-pentetreotide (96% vs. 72%; 94% vs. 82%, respectively) [68], suggesting its pivotal role in NETs clinical practice. [^68^Ga]-DOTA-TATE was also correlated to [^18^F]-FDG in patients with gastro-entero-pancreatic neuroendocrine tumors (GEP-NETs), showing differential uptake of two radiopharmaceuticals in primary and metastatic lesions in relation to the tumor grading. These findings confirmed the utility of combining [^68^Ga]-DOTA-TATE and [^18^F]-FDG PET/CT in intermediate-grade GEP-NETs to help the therapy decision, leading to changed management in 59% of enrolled patients [75]. Recently, DOTA-TATE was radiolabeled with ^64^Cu and its diagnostic performance was compared to [^68^Ga]-DOTA-TOC uptake in NETs patients [76,77].

However, other studies reported that [^68^Ga]-DOTA-TOC affects the treatment decision in a range of 38–59.6% [78,79,80], with higher sensitivity than ^111^In-pentetreotide (99.9% vs. 60%) [81], playing a significant role in tumor’s therapy response also in patients with GEP-NETs who received PRRT [82]. Furthermore, [^68^Ga]-DOTA-TOC uptake significantly well correlated with SSTR2 and Ki-67 gene expression in NEC, evaluated by real-time polymerase chain reaction (qPCR), opening the doors to a possible dual-targeting therapy with mTOR-inhibitor and SST-analog [83].

[^68^Ga]-DOTA-TOC was also compared to [^18^F]-FDG uptake in the initial stage and follow-up of patients with histological confirmation of NETs and treated with PRRT, showing that patients may develop lesions positive to [^18^F]-FDG during follow-up. These results suggested the combination of [^68^Ga]-DOTA-TOC and [^18^F]-FDG PET/CT in the long-term follow-up of NETs patients, particularly when disease progression occurs [84] although this approach is still not universally recognized in routine practice [85]. Moreover, TOC peptide has been radiolabeled with ^99m^Tc ([^99m^Tc]-Hynic-TOC) for imaging of midgut NETs in 31 patients, 13 for primary staging and 18 for follow-up. The double acquisition at 1 and 4 h post radiopharmaceutical injection allows a reduced risk of false findings in SSTR-positive lesions with higher sensitivity, image quality and lower radiation exposure for patients in comparison to [^111^In]-octreotide [86].

An SST2 antagonist, called JR11, has been conjugated with NODAGA and radiolabeled with ^68^Ga, producing [^68^Ga]-OPS202 that has been investigated in an open-label, microdosing study to evaluate the safety, biodistribution, dosimetry and preliminary efficacy of radiopharmaceutical for imaging of SSTR-positive GEP-NETs patients (ClinicalTrials.gov Identifier: NCT02162446). Experimental data resulted in rapid clearance from blood, low background noise in liver and gut, good detection of metastatic lesions and comparable dosimetry data to [^68^Ga]-DOTA-peptides, although further studies are needed [87].

The expression of SSTR 2 and 5 was analyzed in circulating tumor cells (CTC) from patients with G1 or G2 NETs whose SSTR expression was confirmed by PET/CT with [^68^Ga]-DOTA-TATE. CTC was detected in 68% of patients, of which 33% expressed SSTR2 or SSTR5 with a prevalently membranous distribution within the cells, indicating an intra-patient heterogeneous SSTR expression that might reflect the prognosis and the management of a single patient (ClinicalTrials.gov Identifier: NCT02075606) [88].

Actually, several clinical trials are in progress to test the safety and efficacy of PRRT with ^177^Lu or ^90^Y-radiolabeled somatostatin analogs in metastatic patients, e.g., NETTER-1 trial (ClinicalTrials.gov Identifier: NCT01578239) [89,90,91].

### Imaging Treatment Response

As mentioned before, RECIST 1.1 criteria are mainly utilized for therapy monitoring and follow-up in the oncological field [22], but applying RECIST to NETs is slightly limited because of tumor characteristics (slow growth, cystic formations) and available targeted therapies (e.g., everolimus) that lead to a disease stabilization rather than tumor volume reduction [92]. In 2007, Choi et al. proposed novel criteria to evaluate tumor volume and density, especially in gastrointestinal stromal tumors (GISTs) patients treated with imatinib, demonstrating a higher response rate than RECIST (80% vs. 43%) and more predictive of time to tumor progression and overall survival [93,94,95]. The comparison between RECIST 1.1 and Choi criteria has been recently applied to evaluate the early response in patients with advanced GEP-NETs and treated with sunitinib and in patients with pancreatic NETs (ClinicalTrials.gov Identifier: NCT02841865) [96].

**Figure 4:**
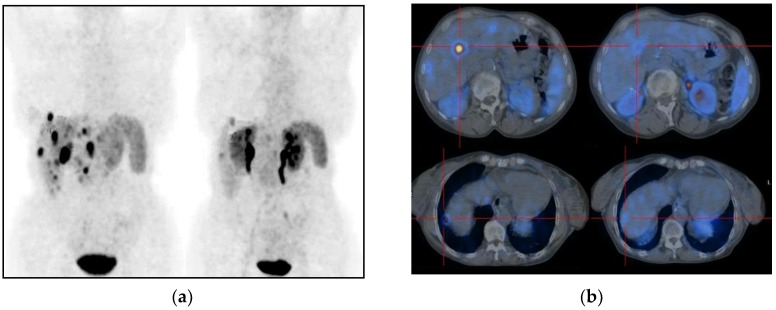
Example of [^68^Ga]-DOTA-NOC PET/CT before and after treatment with somatostatin analogs in a patient with metastatic ileal neuroendocrine tumors (NET). (**a**) Multi intensity projection (MIP) views of several liver metastases at staging (left) and after treatment (right); (**b**) transaxial images of liver showing the presence of metastasis (left) and their almost complete disappearance after treatment.

Very recently, Chan and colleagues introduced a new grading score, the NETPET grade, dividing metastatic NETs patients with certain histological diagnosis into five categories (P0-P5) in relation to lesions characteristics, especially the avidity for [^18^F]-FDG or SSTR and, thus, integrating the imaging results of [^18^F]-FDG and SSTR PET/CT. The statistical analysis showed a significant correlation between the NETPET grade and WHO 2010 histological grade, proposing the NETPET scoring system as a prognostic factor for improving the NETs patients’ management [97]. An example of [^68^Ga]-DOTA-NOC PET/CT before and after treatment with somatostatin analogs is shown in Figure 4.

**Figure 5:**
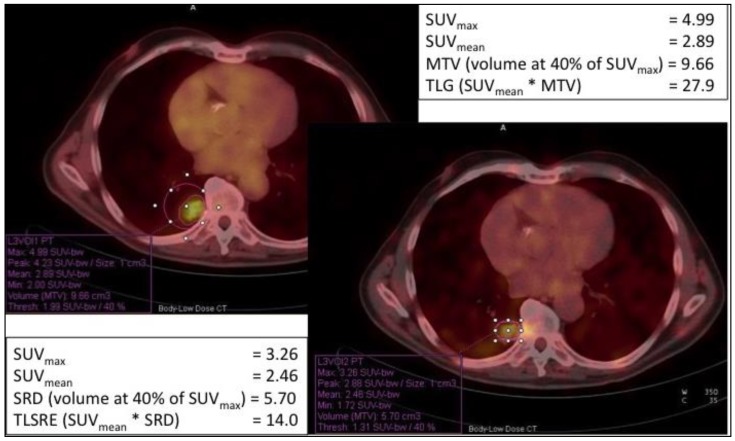
New evaluation prognostic parameters for NETs comparing [^18^F]-FDG PET/CT (left image) and [^68^Ga]-DOTA-NOC imaging (right image). The same lesion was analyzed by both [^18^F]-FDG and [^68^Ga]-DOTA-NOC, measuring SUV_max_, SUV_mean_ for both PET scans, MTV and TLG for [^18^F]-FDG, SRD and TLSRE for [^68^Ga]-DOTA-NOC. Considering the same volume of lesion, TLG and TLSRE values reported differential expression of SSTR and glucose transporters (GLUT) in the lesion. SUV = Standard Uptake Value; MTV = Metabolic Tumor Volume; TLG = Total Lesion Glycolysis; SRD = Somatostatin Receptor Density; TLSRE = Total Lesion Somatostatin Receptor Expression.

Moreover, other studies proposed new volumetric and metabolic parameters that might provide additional information on treatment response and monitoring, combining [^18^F]-FDG and SSTR PET/CT [98,99]. In addition to SUV_max_ and SUV_mean_, the included parameters are Metabolic Tumor Volume (MTV) and Total Lesion Glycolysis (TLG) for [^18^F]-FDG, Somatostatin Receptor Density (SRD) and Total Lesion Somatostatin Receptor Expression (TLSRE) for [^68^Ga]-DOTA-peptides. Considering MTV and SRD as the volume at 40% of SUV_max_, it is possible multiplying MTV or SRD with SUV_mean_ to obtain TLG and TLSRE, respectively. Measuring these parameters for each lesion, these allow an in vivo non-invasive evaluation of glucose transporters (GLUT) and SSTR expression in the same lesion (Figure 5).

## 4. Conclusions

In this review, we provided an overview of several different radiopharmaceuticals and their use for imaging in two specific cancer types, namely breast cancer and NETs (Table 1). However, this concept can be used for a variety of oncological diseases.

The concept of the organism as a molecular entity is increasingly getting a foothold with the aim to understand which molecular pathways and mechanisms are involved in the oncological diseases. Molecular imaging has really contributed to explore which target molecules, such as membrane/cytoplasmic receptors or cytokines, have an essential role in the pathogenesis and progression of diseases through the development of different radiopharmaceuticals.

Nuclear medicine, combined with radiological modalities, provide anatomical and functional information in a non-invasive manner and allow a target localization and quantification, guiding the choice of the most appropriate therapy for each patient and predicting its efficacy. Moreover, this strategy avoids the use of unnecessary drugs influencing therapy decision making and reducing the costs of patients’ management, particularly due to the expensive available therapies like MoAbs.

The need for prospective studies is of great importance to translate the experimental therapies to clinical practice, seeking personalized therapy not only for individual subjects, but also for single lesions within the individual patient. Thus, NM techniques might perform a key role in clinics, including the diagnosis, prognosis, treatment decision and efficacy monitoring.

## Figures and Tables

**Table 1:** Overview of discussed radiopharmaceuticals.

Radiopharmaceutical	Indication
[^18^F]-FES	Breast Cancer
[^18^F]-FDHT	Prostate and breast cancer
[^111^In]/[^89^Zr]-trastuzumab	Breast Cancer
[^68^Ga]-DOTA-TOC/TATE/NOC	Neuroendocrine tumors
[^68^Ga]-DOTAGA-TATE/TOC	Neuroendocrine tumors
[^68^Ga]-OPS202	Neuroendocrine tumors
[^99m^Tc]-Hynic-TOC	Neuroendocrine tumors
[^18^F]-FES	Breast Cancer
[^18^F]-FDHT	Prostate and breast cancer
[^111^In]/[^89^Zr]-trastuzumab	Breast Cancer
[^68^Ga]-DOTA-TOC/TATE/NOC	Neuroendocrine tumors

[^18^F]-FES = 16α-[^18^F]-fluoro-17β-estradiol; [^18^F]-FDHT = fluorodihydrotestosterone; [^68^Ga]-DOTA-Tyr-3-octreotide = DOTA-TOC; [^68^Ga]-DOTA-NaI-octreotide = DOTA-NOC; [^68^Ga]-DOTA-octreotate = DOTA-TATE; OPS202 = NODAGA-JR11.
